# Domain shuffling of endolysin LysKB317 to control *Limosilactobacillus fermentum* in corn mash fermentation

**DOI:** 10.1016/j.btre.2026.e00973

**Published:** 2026-07-19

**Authors:** Shao-Yeh Lu, Zhengliang L. Wu, Moses Y. Martinez, Maulik H. Patel, Christopher D. Skory

**Affiliations:** a USDA, Agricultural Research Service, National Center for Agricultural Utilization Research, Renewable Product Technology Research Unit, Peoria, IL, 61604, USA; b Oak Ridge Institute for Science and Education (ORISE), Oak Ridge, TN, 37830, USA

**Keywords:** Endolysin, Fuel ethanol, Antimicrobial, Domain shuffle, Corn mash fermentation

## Abstract

Lactic acid bacteria (LAB), such as *Limosilactobacillus fermentum* are persistent contaminants in bioethanol fermentation facilities and inhibit *Saccharomyces cerevisiae*, reducing ethanol yields. Rising resistance to antibiotics such as virginiamycin makes contamination control increasingly difficult. Recombinant endolysins such as LysKB317 provide a promising alternative. To improve LysKB317 performance, five variants were engineered by duplicating and rearranging its enzymatically active (EAD) and cell binding (CBD) domains. These constructs were compared to the wild-type endolysin. Differential scanning calorimetry (DSC) and bacterial lytic assays demonstrated that domain shuffling altered bacteriolytic activity and thermostability. The wild-type LysKB317 exhibited the highest initial lytic activity (1.16 × 10^-4^ OD/s‧µM), whereas LysKB317_EADx3-CBD_ displayed comparable bacteriolytic kinetics (7.99 × 10^-5^ OD/s‧µM) and LysKB317_CBD-EAD-CBD_ (ΔCp = 24.66 kJ/mol‧K) exhibited the greatest thermostability. Despite reduced activity, both variants effectively control LAB contaminants and prevented stalled corn mash fermentation. Domain shuffling demonstrated effective bacterial control strategy for generating novel endolysins for biocontrol.

## Highlights

• Catalytic and binding domain shuffling generated novel endolysins.
• Domain-shuffled variants effectively reduce bacterial contamination in corn mash.
• Domain shuffling strategy accelerates creation of novel endolysins for biocontrol.

## 1 Introduction

Endolysins, known as peptidoglycan hydrolases, lysins or enzybiotics are bacterial cell wall-degrading enzymes encoded by bacterial targeting viruses (bacteriophages or phages). These enzymes play a crucial role in the end stage of the phage replication cycle by hydrolyzing the peptidoglycan layer of the bacterial cell wall, leading to host cell lysis and release of phage progeny [1]. The process is facilitated by holins, phage-encoded membrane proteins that create pores in the cytoplasmic membrane, thus enabling endolysins to access and degrade the peptidoglycan from within [1,2].

Phage-based antibacterial strategies have been explored in several agricultural and biomedical settings [1,[3], [4], [5], [6]]. However, limited studies have been conducted to control bacterial contamination present in bioethanol fermentation facilities [[7], [8], [9], [10]]. The application of bacteriophages as a bacterial contaminant mitigation strategy is often constrained by the phages’ narrow host range and the potential for the development of bacterial resistance [11]. Some mechanisms of resistance include receptor mutations [12], cell surface structure modifications, and Clustered Regularly Interspaced Short Palindromic Repeats (CRISPR) Cas system [13]. In contrast, phage-derived proteins, such as endolysins, have demonstrated greater efficacy in targeting bacterial strains while posing a lower risk of resistance development. This strategy is particularly useful in combating the growing threat of antibiotic-resistant bacterial contamination [[14], [15], [16]].

The molecular structure of Gram-positive bacteria targeting endolysins generally consists of modular domains that comprise an enzymatically active domain (EAD) and a cell-binding domain (CBD) joined by a short flexible linker [17,18], in contrast to a typical single globular catalytic domain of Gram-negative bacterial targeting endolysins [19]. Several studies have investigated the modularity of endolysins by generating chimeric enzymes through domain shuffling, typically involving the swapping of EADs or CBDs from different endolysins for the development of improved antimicrobial therapeutics [[20], [21], [22], [23], [24], [25], [26], [27], [28], [29]]. However, the intramolecular domain shuffling of a single endolysin remains largely unexplored.

Our research group developed a novel sustainable strategy for mitigating lactic acid bacteria (LAB) contamination [[30], [31], [32]]. This approach utilizes endolysins derived from *Lactobacillus* bacteriophages (e.g., LysA, LysA2, LysKB317; [8,33]) to specifically target Gram-positive *Lactobacillus* species. These enzymes possess a modular structure comprising an N-terminal glycoside hydrolase (GH25) family-like EAD and a C-terminal bacterial Src homology 3 (SH3b) -like CBD [34], which specifically targets *Limosilactobacillus fermentum*, a known bacterial contaminant that inhibits *Saccharomyces cerevisiae* fermentation, resulting in stalled corn ethanol fermentation [32,33]. Here, we explored different variant designs based on endolysin LysKB317 and examined their bacteriolytic activities against *L. fermentum*.

Given the binding specificity of LysKB317 against *L. fermentum*, we hypothesized that increasing the tandem repeats of the CBD domain would enhance the binding affinity of the endolysin to the target cells, and similarly, an increase in tandem repeats of the EAD would alter the bacteriolytic kinetics. To test this, endolysin variants were designed with one, two, or three copies of the EAD or CBD arranged in tandem or flanking configurations, while the wild-type LysKB317 (LysKB317_EAD-CBD_) contained single copy of EAD and CBD, served as the control. Bacteriolytic activity under corn mash fermentation conditions was evaluated to determine if domain multiplicity and orientation shuffling improved performance. Results showed that domain shuffling altered bacteriolytic kinetics and thermostability while maintaining effective bacterial control during corn mash fermentation.

Here, we showed that endolysin LysKB317 containing three EAD tandem repeats (LysKB317_EADx3-CBD_) exhibited bacteriolytic kinetics comparable to the wild-type (one EAD) while maintaining effective bacterial control and biofilm reduction. Flanking domain arrangements such as LysKB317_CBD-EAD-CBD_ did not improve the bacteriolytic activity of the construct, but they improved its thermostability. In addition, LysKB317_EAD_ and LysKB317_CBD_ exhibited little to no detectable activity against contaminants as single-domain controls. Overall, we demonstrated that rearranging modular domains from the same endolysin alters bacteriolytic kinetics and thermostability without compromising bacteriolytic activity during corn mash fermentation.

## 2 Results

**Fig. 1:**
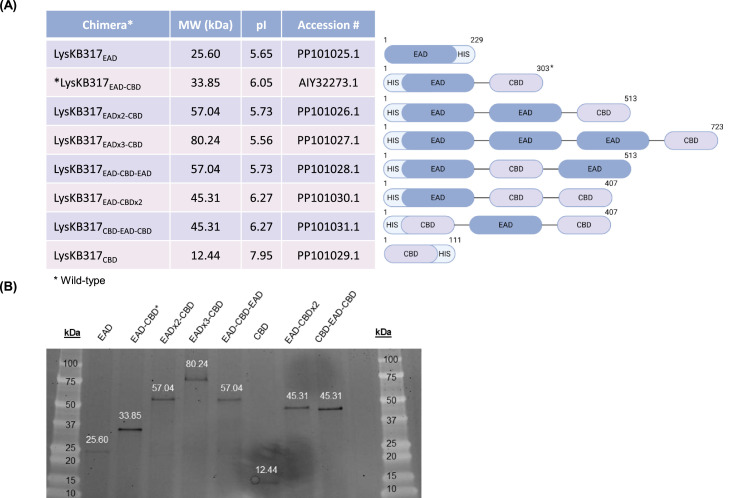
(**A**) Schematic representation of endolysin LysKB317 variant constructs used (not drawn to scale). Enzymatically active domain (EAD) is a glycosyl hydrolases 25 family (GH25)-like domain. Cell-wall binding domain (CBD) consists of a SH3b-like family domain. Domain shuffled constructs utilized in this research consist of hexa-histidine-tag in the N-terminus of EAD (LysKB317_EADx3-CBD_, LysKB317_EADx2-CBD_ and LysKB317_EAD-CBD-EAD_) and CBD (LysKB317_CBD-EAD-CBD_). Single modular domain constructs (EAD and CBD) consist of C-terminus hexa-histidine tag. Amino acid length of variant construct, molecular weight (MW), isoelectric point (pI) and protein accession numbers are noted next to the schematic representation. (**B**) Validation of His_6x_-tagged purified domain shuffled variant enzymes were performed using SDS-PAGE. Molecular weight (kDa) of each enzyme labeled as EAD (LysKB317_EAD_), EAD-CBD* (LysKB317 wild-type), EADx2-CBD (LysKB317_EADx2-CBD_), EADx3-CBD (LysKB317_EADx3-CBD_), EAD-CBD-EAD (LysKB317_EAD-CBD-EAD_), CBD (LysKB317_CBD_), EAD-CBDx2 (LysKB317_EAD-CBDx2_) and CBD-EAD-CBD (LysKB317_CBD-EAD-CBD_). Asterisk (*) represents the wild-type LysKB317.

**Molecular weight and predicted isoelectric point (pI) of domain shuffled endolysins**. The predicted molecular weight, isoelectric points (pIs; ExPASy [35]) and the schematic representation of each LysKB317domain-shuffled variants are shown in Fig. 1A **together with their corresponding GenBank accession numbers**. As expected, the molecular weight increased with additional EAD or CBD domain repeats, whereas the predicted pIs of constructs containing at least one EAD remained within a narrow acidic range (5.56 to 6.27). On the other hand, the CBD-only construct exhibited a higher predicted pI (7.95). The molecular weight of purified recombinant endolysins were verified by sodium dodecyl sulfate-polyacrylamide gel electrophoresis (SDS-PAGE; Fig. 1B). AlphaFold 3 [36] was utilized to predict the tertiary structures of individual domains (EAD and CBD) and all other endolysin variants (Fig. S1).

**Fig. 2:**
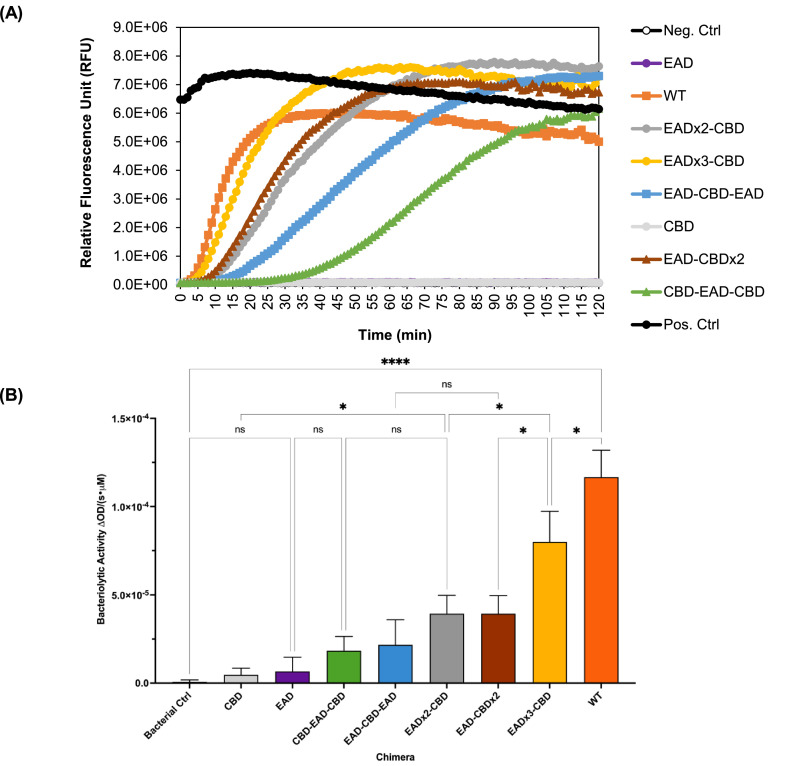
**(A)** LysKB317 variant constructs undergone bacteriolytic assay using a SYTOX Green nucleic acid stain. Lysis of *L. fermentum* 0315-25 for 120 min at room temperature and measured the relative fluorescence units (RFU) with 96-well plate reader at 485/535 nm (Ex/Em). Positive control bacterial cells pretreated with isopropanol were used (Pos. Ctrl; black circle). Negative control in wells containing *L. fermentum* were designated as bacterial control (Neg. Ctrl; white circle). All purified enzyme constructs were standardized to 0.01 µM and were added to target cells in triplicates on a clear flat bottom 96-well plate. Wild-type endolysin LysKB317 (WT; orange square), LysKB317_EADx3-CBD_ (EADx3-CBD; yellow circle), LysKB317_EAD-CBDx2_ (EAD-CBDx2; dark red triangle), LysKB317_EADx2-CBD_ (EADx2-CBD; grey circle), LysKB317_EAD-CBD-EAD_ (EAD-CBD-EAD; blue square), LysKB317_CBD-EAD-CBD_ (CBD-EAD-CBD; green open triangle), LysKB317_EAD_ (EAD; purple circle), and LysKB317_CBD_ (CBD; light grey circle) were all performed in three independents biologically replicates (*n* = 3). Due to minimal to no measurable bacteriolytic activity throughout the assay, curves from LysKB317_EAD_ (EAD) and negative control are masked by the LysKB317_CBD_ (CBD). **(B)** Bacteriolytic activity (BLA) of individual domains and domains shuffled variants of LysKB317. Data are reported in OD/(s‧µM). Three independent replicates (*n* = 3); error bars indicate standard deviation (SD). Statistical significance (*p < 0.05, ****p < 0.0001, and ns = no significance).

**SYTOX Green fluorescence kinetics of domain-shuffled endolysin variants.** The bacteriolytic activities of the domain-shuffled endolysins variants were compared using the SYTOX Green fluorescence stain (Fig. 2A) described in the bacteriolytic assay (BLA). The positive control (chemically killed cells; black circle) plateaued at approximately 14 min with a 7.5 × 10^6^ RFU while the negative control (cells without endolysin; white circle) only maintained a minimal average RFU of 8.8 × 10^4^ throughout the course.

Among the domain-shuffled constructs, EADx3-CBD (gold circle) and EADx2-CBD (gray circle) displayed rapid initial increases in RFU values, reached peak values of approximately 7.8 × 10^6^ and 7.9 × 10^6^ RFU and plateaued at approximately 82 min and 91 min. The EAD-CBDx2 (brown triangle) and CBD-EAD-CBD (green triangle) constructs demonstrated intermediate fluorescence signals, with peak RFU values reaching approximately 7.2 × 10^6^ at 92 min and 6.2 × 10^6^ at 118 min, respectively. The EAD-CBD-EAD (blue square) construct also reached similar fluorescence intensity (7.4 × 10^6^ RFU), but with slower kinetics (115 min; Fig. 2A). The wild-type LysKB317 (WT; orange square) construct produced a lower cumulative fluorescence signal (6.1 × 10^6^ RFU) and earlier plateau (44 min), than the engineered variants. The CBD-only (light gray circle; 1.0 × 10^5^ RFU) and EAD-only (purple circle; 1.1 × 10^5^ RFU) constructs resulted in minimal fluorescence values similar to the negative control (8.8 × 10^4^ RFU), confirming that the CBD-only and EAD-only constructs do not contribute significantly to bacteriolytic activity. The SYTOX Green dye provides an indirect measure of bacteriolysis through cumulative nucleic acid release following membrane permeabilization, the fluorescence profiles primarily represent cumulative fluorescence over time rather than direct measurements of endolysin activity.

To quantitatively compare the initial lytic kinetics of the engineered endolysin variants, bacteriolytic activity rate (BLA; Eq. (1)) was calculated from the early linear phase of the reaction (Fig. 2B). The wild-type showed the highest initial lytic rate of 1.16 × 10^-4^ OD/s•µM and was significantly (*p* < 0.0001) greater than all engineered variants including individual domains (EAD and CBD; Fig. 2B) with the exception of LysKB317_EADx3-CBD_ (7.99 × 10^-5^ OD/s•µM; *p < 0.0252). LysKB317_CBD-EAD-CBD_, LysKB317_EAD-CBD-EAD_, LysKB317_EADx2-CBD_, and LysKB317_EAD-CBDx2_ exhibited significantly lower initial lytic rates than LysKB317 (wild-type). The isolated EAD and CBD domains displayed minimal bacteriolytic activity comparable to the bacterial control (negative control). Although EAD alone exhibited slightly higher bacteriolytic activity than CBD alone; however, the differences were not statistically significant (*p* > 0.999). Similar trends were observed when the initial bacteriolytic rates were calculated from changes in fluorescence (RFU) values (Fig. S2).

**Fig. 3:**
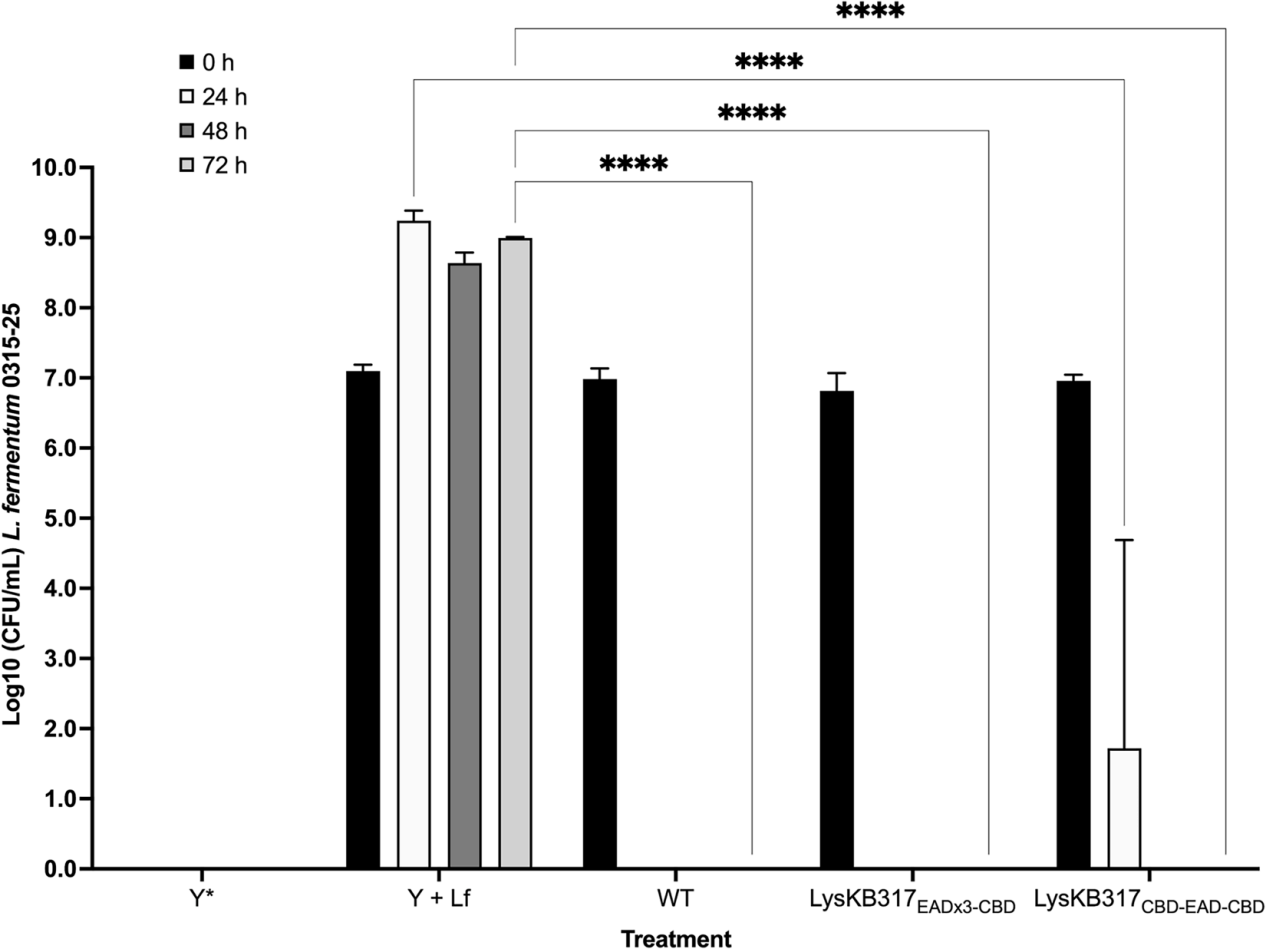
**Bacterial colony forming unit count of LysKB317 variants against *L. fermentum* in corn mash fermentation.** Yeast only control (Y*) showed below detectable CFU/mL. Yeast sample challenged with bacteria (Y + Lf); Yeast with bacteria with LysKB317 wild-type treatment (WT); yeast and bacteria with LysKB317_EADx3-CBD_ treatment (EADx3-CBD) and yeast with bacteria plus LysKB317_CBD-EAD-CBD_ treatment (CBD-EAD-CBD). Measurement taken at 0 h (black), 24 h (white), 48 h (dark grey), and 72 h (light grey). Data were measured at Log(CFU/mL) at *n* = 3 with 3 independent biological replication and error bars representing standard deviation (SD). Statistical significance (****p < 0.0001).

**Domain-shuffled constructs mitigated bacterial contamination in corn mash fermentation for 72 h**. Two of the five endolysin LysKB317 variants (LysKB317_EADx3-CBD_, LysKB317_CBD-EAD-CBD_) were selected to represent the engineered domain-shuffled constructs variants with the highest initial bacteriolytic kinetics (7.99 × 10^-5^ OD/s‧µM) and lowest bacteriolytic kinetics (1.80 × 10^-5^ OD/s‧µM) compared to the wild-type LysKB317 (WT; 1.16 × 10^-4^ OD/s‧µM; Fig. 2). Over a period of 72 h, contaminated corn mash samples in each treatment condition were sampled every 24 h and plated to enumerate the bacterial load (Fig. 3). No *L. fermentum* was detected (< 1 log CFU/mL) in yeast only flask (Y*). While samples from yeast with *L. fermentum* showed a sustained bacterial load of approximately log 7.1 to 9.0 CFU/mL over 72 h (Y + Lf). Samples treated with the wild-type (Y + Lf + LysKB317; *p* < 0.0001) and variant endolysin (Y + Lf + LysKB317_EADx3-CBD_; ****p < 0.0001) both showed bacterial levels below the detectable limit (< 1 log CFU/mL) after 24 h and remained undetectable for the rest of fermentation. Contaminated corn mash samples treated with LysKB317_CBD-EAD-CBD_ (Y + Lf + LysKB317_CBD-EAD-CBD_) exhibited an approximately 5-log reduction in bacterial load (1.72-log CFU/mL) after the first 24 h (6.96-log CFU/mL) and continued to decrease to an undetectable level past 24 h reached the detection limit during the remainder of the 72 h fermentation.

**Fig. 4:**
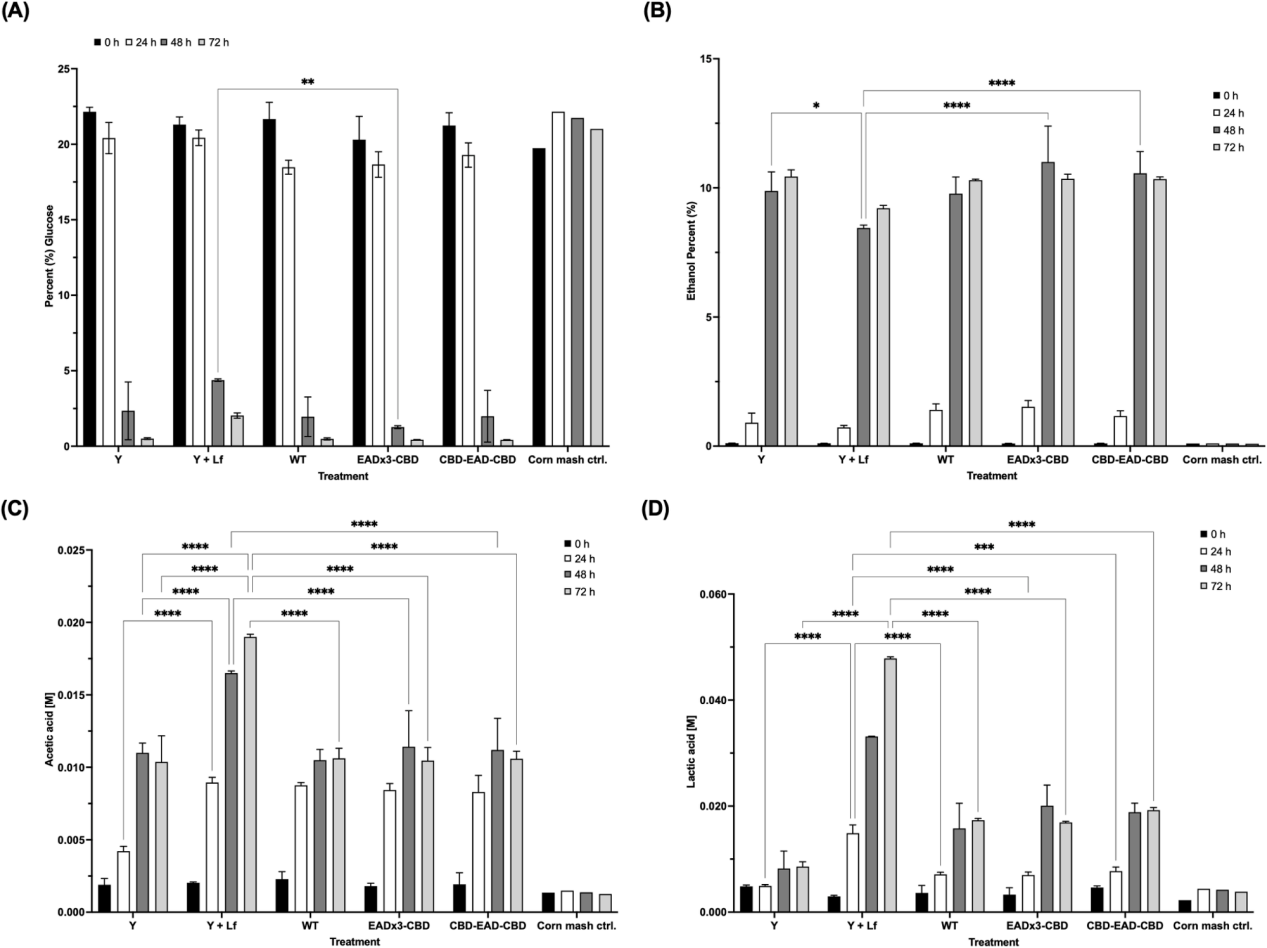
**HPLC analysis of fermentation metabolites of 72 h corn mash fermentation**. (A) Glucose and (B) ethanol are defined as [g/100 mL] and are referred to as percent (%) w/v. (C) Acetic acid and (D) lactic acid are referred to as molar [M]. Three independent biological replication (*n* = 3) and error bars representing standard deviation (SD). Statistical significance (*p < 0.05, **p < 0.01, and ****p < 0.0001).

**Analysis of glucose, ethanol, and organic acids by high-performance liquid chromatography (HPLC) of domain-shuffled endolysin treated corn mash samples.** To examine the glucose utilization, ethanol, acetic, and lactic acid production in corn mash fermentation flasks, samples were obtained every 24 h for three days and analyzed by HPLC (Fig. 4). Residual percent glucose levels (v/w) steadily decreased from 0 h to 72 h for yeast control (Y) and all WT (LysKB317_EAD-CBD_) and variant treatments (LysKB317_EADx3-CBD_ and LysKB317_CBD-EAD-CBD_) at 0.42 – 0.49% compared (ns; *p* > 0.9999)to the untreated bacterial infection control (2.03 %; *p* = 0.783, *p* = 0.733 and *p* = 0.721 respectively; Fig. 4A).

Ethanol production in endolysin treatment samples did not differ significantly between the wild-type (WT) and the domain-shuffled enzymes (LysKB317_EADx3-CBD_; *p* > 0.999, and LysKB317_CBD-EAD-CBD_; *p* > 0.999) and yeast-only control (ns; *p* > 0.999). All three conditions produced approximately 10.3% w/v ethanol at the end of 72-h fermentation. Significant differences (****p < 0.0001) in ethanol production were observed when treatment groups (WT; *p = 0.0363 or variant endolysins; ****p < 0.0001) were compared with the untreated infection control at 48 h (8.4 % w/v; Fig. 4B).

The level of accumulated acetic acid in the corn mash samples throughout fermentation remained stable, ranging from 0.0104 M to 0.0106 M for the wild-type and variant endolysins with no significant differences among treatments (ns; *p* > 0.05). A significant difference (*p* < 0.0001) was observed when treatment groups were compared with the untreated contamination control (Fig. 4C). Similar trends were observed for the accumulation of lactic acid. The level of lactic acid concentration was slightly lower at 0.0086 M (*p < 0.05) for yeast only sample compared to all three endolysin treatment conditions at 0.0174 M (WT), 0.0169 M (LysKB317_EADx3-CBD_) and 0.0193M (LysSKB317_CBD-EAD-CBD_) at the end of e fermentation (Fig. 4D). No significant differences were observed when comparing treatment conditions (ns; *p* > 0.999). A significant reduction in lactic acid was observed when the treatment conditions were compared with the untreated control (Y + Lf; 0.0479 M; ****p < 0.0001).

**Fig. 5:**
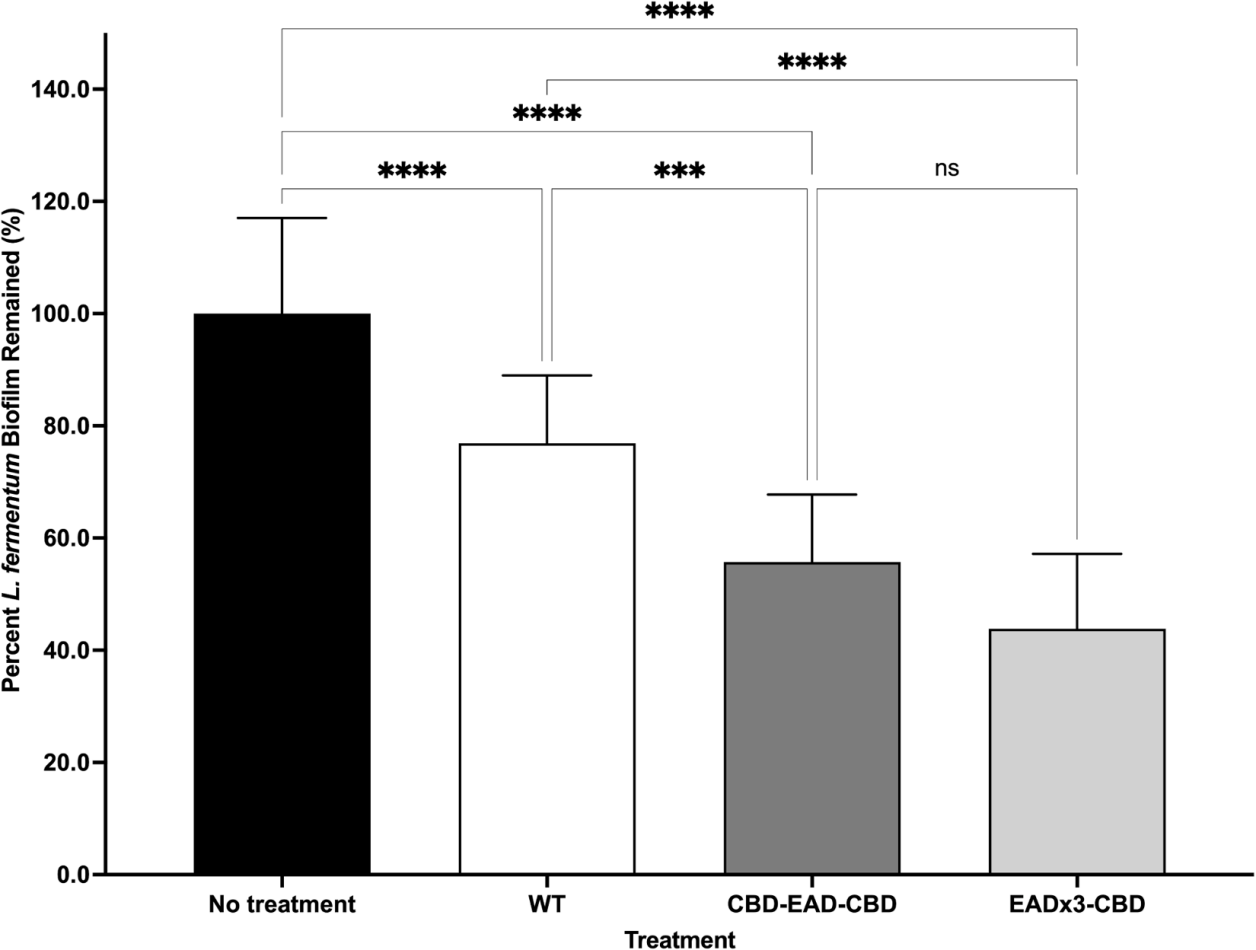
Biofilm reduction assay of LysKB317 domain shuffled variants. Bars represent averaged percent *L. fermentum* 0315-1B biofilm remaining after 20 µM purified endolysin treatment for 60 min at room temperature. Assays were repeated with fifteen independent biological repeats (*n* = 15) where error bars are standard deviation (SD). Statistical significance (***p < 0.001 and ****p < 0.0001; ns = no significance).

**Endolysin LysKB317 and domain-shuffled variants reduce biofilm.** *L. fermentum* 0315-1B**,** a biofilm producing strain isolated from a bioethanol facility, was used to generate mature biofilm for 10 days prior to treatment. The biofilm was treated with purified recombinant endolysin LysKB317 and the engineered variants (LysKB317_EADx3-CBD_ and LysKB317_CBD-EAD-CBD_) at 20 µM for 60 min at room temperature (Fig. 5). Compared with the untreated control (No treatment; 0 % biofilm biomass reduction), all endolysin treatments significantly reduced biofilm biomass (****p < 0.001). The LysKB317 (WT) reduced 23.1% (****p < 0.0001) biofilm biomass, while the variants (LysKB317_EADx3-CBD_ and LysKB317_CBD-EAD-CBD_) significantly reduced biofilm biomass by 56.2 % (****p < 0.0001) and 44.3 % (****p < 0.0001), respectively (Fig. 5). No significant differences were observed between the two engineered endolysin variants (ns; *p* = 0.068).

**Differential Scanning Calorimeter (DSC) analysis of thermostability in domain shuffled variant.** A differential scanning calorimeter (NanoDSC) was used to evaluate the thermostability and unfolding properties of endolysin variant constructs containing tandem repeats or flanking domain arrangement constructs (LysKB317_EAD-CBD-EAD_ or LysKB317_CBD-EAD-CBD_). The combination of transition temperature (T_max_), enthalpy change (ΔH), entropy change (ΔS), heat capacity change (ΔCp), full width at half maximum (FWHM), and unfolding area was accessed to determine if enzyme improvements were made when compared to the wild-type (Table 3). The heat capacity change upon thermal unfolding provided crucial insights into protein conformational flexibility and the extent of surface exposure of hydrophobic residues during denaturation.

Among all tested constructs, LysKB317_CBD-EAD-CBD_ exhibited the highest thermal stability, characterized by the highest ΔCp value of 24.66 kJ/mol‧K and ΔH (1900.08 kJ/mol). The flanking construct also had an unfolding area under the DSC curve after Gaussian fit values adjustment of 1,887.33 with the highest amplitude value of 610.93 and the lowest FWHM value (2.90 °C) indicating a sharp, cooperative transition with T_max_ at 62.67 °C (Table 3). On the contrary, the flanking construct of LysKB317_EAD-CBD-EAD_ exhibited the weakest performance across all parameters, including the lowest T_max_ (54.44 °C) and ΔH (141.11 kJ/mol). This construct resulted in a lower ΔCp value of 2.16 kJ/mol‧K and unfolding cooperativity (2.95 °C).

The second and third variants (LysKB317_EAD-CBDx2_ and LysKB317_EADx3-CBD_) demonstrated enhanced stability with ΔH of 1,400.53 kJ/mol and 865.48 kJ/mol, respectively. Their moderate ΔCp values of 18.07 kJ/mol‧K and 12.16 kJ/mol‧K, along with T_max_ 62.99°C and 58.61°C, indicated a marginally lower overall thermodynamic profile.

The wild-type endolysin, LysKB317_EAD-CBD_, displayed a baseline thermodynamic profile with a modest T_max_ of 62.78 °C, ΔH of 777.69 kJ/mol, and a ΔCp value of 10.07 kJ/mol‧K with a moderate amplitude (224.24) and an area value of 779.60. This was followed by the variant LysKB317_EADx2-CBD_ that had ΔH (420.97 kJ/mol), T_max_ (60.07 °C), and ΔCp (5.74 kJ/mol‧K) suggesting a less thermodynamically architecture. Individual EAD and CBD domains showed limited change in heat capacity (1.87 and 2.15 kJ/mol‧K) and high T_max_ values of 64.76°C and 67.12°C, respectively, but significantly lower ΔH (149.53 and 179.76 kJ/mol) indicating lower unfolding cooperativity (Table 3).

**Fig. 6:**
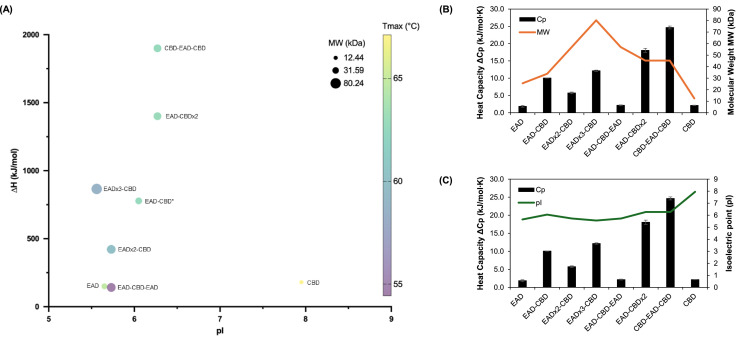
**Differential scanning calorimetry analysis of LysKB317 variants (A).** Thermodynamic stability (ΔH) versus isoelectric point (pI) multi-variable chart with endolysin molecular weight (MW; kDa) represented with different diameter of black circle and color gradient represents transition temperature at 50% dissociation of endolysin variants in T_max_ (°C). **(B).** Change in heat capacity (ΔCp) and molecular weight (MW). **(C).** Change in heat capacity (ΔCp) and isoelectric point prediction values for the variants (pI). Average change in heat capacity (ΔCp) was calculated from three independent biological repeats (*n* = 3) of NanoDSC samples from each endolysin variant with error bars as standard deviation (SD).

The relationship between isoelectric point (pI) and unfolding enthalpy (ΔH) was examined with bubble size corresponding to molecular weight (MW) and color gradient scale indicating T_max_ (Fig. 6A). The thermostability results indicated that the endolysin LysKB317_CBD-EAD-CBD_ flanking variant was the most thermally stable construct. Whereas the LysKB317_EAD-CBD-EAD_ flanking variant was the least thermally stable. Changes in heat capacity (ΔCp) did not correlate with the molecular size (MW) or isoelectric point (pI) of the endolysin constructs (Fig. 6B and C).

## 3 Discussion

Gram-positive endolysins feature a multi-domain architecture [23,37,38] that enables researchers to design novel endolysins to broaden the range of targeted bacterial species and/or enhance bacteriolytic activity. The modularity of the enzyme enables the rational design of chimeric endolysins through domain shuffling [19,26,37,[39], [40], [41]]. Typically, this involves replacing the domain (EAD or CBD) under study with a domain from a different endolysin, aiming to enhance functionality [28,29,47]. However, to the best of our knowledge, only one study to date has investigated the potential of generating chimeric endolysins by recombining CBD domain derived from the same parental endolysin [42]. This study presents the characterization and comparison of several domain-shuffled designs derived from one single endolysin, LysKB317, with the goal of improving lytic activity, protein stability, and expand our existing repertoire of lactic acid bacteria targeting endolysins for contamination control at bioethanol fermentation facilities. The previously described endolysin LysKB317 (Accession number AIY32273.1) consists of a predicted *N*-acetylmuramidase GH25-like enzymatic activity domain and a predicted SH3b family-like cell-wall binding domain. We previously demonstrated that purified recombinant LysKB317 can mitigate *L. fermentum* contamination and prevent stalled fermentation when exogenously added into corn mash fermentations [33]. The use of recombinant endolysin can be a good alternative to traditional antibiotics. To date, no reports of resistance development have been documented in bacteria treated with recombinant endolysin. However, certain traits in Gram-positive and Gram-negative bacteria can enhance the production of glycol-polymers such as teichoic acid (WTA) and or lipoteichoic acid (LTA), which can reduce endolysin’s bacteriolytic activity [[43], [44], [45]].

**Initial bacteriolytic activity of domain-shuffled endolysins variants.** Our results demonstrated that all five domain shuffled variants of LysKB317 (Fig. 1 and Fig. S1), regardless of domain arrangement, generated higher relative fluorescence units (RFU) values compared to the wild-type in the bacteriolytic assay. This suggests all domain-shuffled variants exhibited gradual and enhanced bacteriolytic effects (Fig. 2A) that outperformed the wild-type in bacteriolytic activity over time (Fig. 2 and Fig. S2). The positive control does not reflect total cell lysis relative to treatments due to DNA loss during preparation through cell washes. The measurements of bacteriolytic activity (BLA; Eq. (1).) and RFU values from individual domains (EAD or CBD) alone resulted in only minimal basal fluorescence signals without statistical significance compared to the bacterial only control (Neg. Ctrl; Fig. 2A). Unlike most Gram-negative targeting endolysins that contain a single domain and some known Gram-positive targeting endolysin EAD domain (e.g., CL1, Jib1 and Endo88) alone demonstrated lytic activity [19,46,47].These results suggest that individual LysKB317 domains (EAD or CBD by itself) do not contribute to bacteriolytic activity, and that effective bacterial cell wall degradation necessitates domain cooperation [[48], [49], [50]]. The BLA activity of negative control and EAD-only curves exhibits similar minimal to no measurable fluorescence signal as the CBD-only curve and are masked by the CBD curve (Fig. 2A).

**Proposed structural model of LysKB317 peptidoglycan (PG) interactions.** The limited activity observed for the isolated EAD and CBD domains suggests that efficient bacteriolysis by endolysin LysKB317 requires cooperation between both domains (Fig. 2B). Based on the AlphaFold 3 structural prediction together with ligand-binding site prediction server such as the PrankWeb [51], and protein-ligand docking prediction (HADDOCK 2.4; [52,53]), we explored a possible structural model for how the EAD and CBD may interact during PG recognition. The predictive computational analysis identified a putative ligand-binding pocket within the posterior region of the CBD (Fig. S3B) and a second pocket within the anterior cavity of the EAD (Fig. S3D). Together, the predicted arrangement of the two domains suggests that the LysKB317 endolysin may adopt a horizontal position along the PG surface (Fig. S3A and E). In this orientation, a central cavity formed between the EAD and CBD could potentially position the substrate for catalysis (Fig. S3A, C, and E). Interestingly, a similar overall domain orientation has been reported for Psm, a *Clostridium perfringens* targeting endolysin, consists of one GH25-like domain EAD and two SH3-like CBD, with structural model derived from X-ray crystallography rather than computational prediction [26]. We include this comparison only to illustrate a possible structural similarity and not to imply a shared catalytic mechanism. It is also important to note that the docking analysis by HADDOCK (Fig. S3F) was performed using a NAG-NAM tetrasaccharide ligand, taken from protein data bank (PDB ID:4PI9 with ligand ID 3LT; [54]), which primarily represents the glycan backbone from PG. Consequently, the model is more informative for GH25-like catalytic domain than for the cell-binding domain such as the SH3b. Predictive interactions of SH3b-like domain may involve additional structural features of the bacterial cell-wall that are not represented in the docking ligand. Although combined computational model provided a plausible hypothesis for the orientation of LysKB317, future structural and biochemical studies such as X-ray crystallography and substrate binding will be required to experimentally validate the proposed binding orientation and cleavage mechanism.

**Domain-shuffled endolysins exhibited bacteriolytic activity comparable to the wild-type LysKB317 in a corn mash fermentation.** To assess the efficacy of endolysin variant constructs under relevant fermentation conditions, LysKB317_EADx3-CBD_ and LysKB317_CBD-EAD-CBD_ were selected based on their calculated bacteriolytic rates (representing the highest and lowest rate, respectively; Fig. 2B and Fig. S2). These constructs were further compared to the wild-type in a bacterial contaminated corn mash fermentation model. Interestingly, despite the contrasting bacteriolytic activity observed (Fig. 2), no significant differences in bacterial viability were detected among the domain-shuffled variants and the wild-type at the end of the fermentation (Fig. 3). However, by the end of the 72-h fermentation, no significant differences in bacterial viability were detected among the wild-type and domain-shuffled variants. This discrepancy may be attributed to inherent limitations of the fermentation model, such as reduced sensitivity and/or limited sampling frequency and sample volume, which can obscure subtle differences of the performance in different endolysin variants. Furthermore, treatment concentration of 0.01 µM could have been in excess and masked the construct specific effects (Fig. 4 and Table 2). Nonetheless, despite differences in the rate of bacterial reduction during the first 24 h, all selected constructs reduced bacterial contamination to below the detection limit by the end of fermentation (72 h), demonstrating that each variant maintained bacteriolytic activity under fermentation conditions. To effectively differentiate subtle differences among different endolysin variants, a continuous monitoring system or scaled-up fermentation model may be required.

**Domain-shuffled LysKB317 variants reduced *L. fermentum* biofilm biomass.** In addition, controlling planktonic bacterial cell growth in corn mash fermentation, domain-shuffled endolysins (LysKB317_EADx3-CBD_ and LysKB317_CBD-EAD-CBD_) also reduces preformed mature biofilm biomass (Fig. 5). Biofilms formed by LAB contaminants are particularly problematic in the bioethanol fermentation facilities because they are persistent, difficult to eradicate, and function as recurrent contamination reservoir [32]. Furthermore, biofilms are challenging to remove using conventional antibiotics [55]. Under the conditions tested (20 µM purified endolysin for 60 min), both endolysin variants (LysKB317_EADx3-CBD_ and LysKB317_CBD-EAD-CBD_) demonstrated greater reduction in biofilm biomass when compared to the wild-type LysKB317. To limit variation associated with crystal violet assay staining, we increased number of biological repeats (*n* = 15). Although, the crystal violet assay measures total biofilm biomass rather than bacterial viability within the biofilm, these findings suggest that domain-shuffling strategies may improve the ability for endolysin LysKB317 to disrupt established biofilms. Nonetheless, more studies are required to determine the strategy effects of biofilm biomass reduction.

**The rate of bacterial lysis in domain shuffled variants did not directly correlate with thermal stability profiles.** Among all variant constructs analyzed by differential scanning calorimetry, LysKB317_CBD-EAD-CBD_ exhibited the highest thermal stability (Table 3 and Fig. 6). The domain arrangement of the enzyme also indicated that the symmetrical display of the CBD may shield the EAD core and contribute to its increased heat capacity (ΔCp). This further suggests that the enzyme is likely to be more stable at higher temperatures [[56], [57], [58]] as confirmed by the elevated T_max_ (Table 3, Fig. 6a, Fig. S4A, and B). Furthermore, multiplicity of CBD domains, both as described above and in tandem arrangements (e.g., LysKB317_EAD-CBDx2_) may result in enhanced intramolecular domain stabilization, which further contributes to the structural rigidity of the enzyme.

**Effect of domain multiplicity on bacteriolytic performance is more prominent with EAD.** While the multiplicity of CBD contributes to increased thermostability of the enzyme, variants with duplicated EADs (LysKB317_EADx2-CBD_ and LysKB317_EAD-CBD-EAD_) showed reduced heat capacity (ΔCp) and T_max_, suggesting a less favorable thermal structural arrangement (Table 3). Nonetheless, constructs containing multiple EADs exhibited higher bacteriolytic activity, on average, than CBD containing variants (Fig. 2 and Fig. S2). Expectedly, constructs containing higher numbers of EADs (e.g., LysKB317_EADx3-CBD_ at pH 5.56) exhibited lower isoelectric points (pIs), which appeared to be beneficial to the domain-shuffled enzymes under corn mash fermentation conditions (pH 4 – 5; [59,60]). This suggests that EAD multiplicity contributes more to the pI shift than CBD multiplicity variants (Table 3) and may support improved enzymatic activity under acidic conditions due to enhanced stability.

**Thermal stability is not merely an indicator of structural robustness**. Importantly, the functional performance of domain-shuffled endolysins is highly dependent on the spatial arrangement of its domain structures. The usefulness of endolysins in industrial applications such as biofuel fermentation depends on enzyme stability under acidic (pH 4–5) and elevated temperature conditions. Constructs exhibiting higher thermal stability and lower isoelectric points (pIs), particularly those containing tandem EADs or flanking CBDs, were more resilient under fermentation conditions. Overall, our constructs support the hypothesis that endolysin LysKB317 containing multiple and strategically arranged CBDs such as LysKB317_CBD-EAD-CBD_ or LysKB317_EAD-CBDx2_ confer overall greater structural stability. In contrast, indiscriminate domain duplications (e.g., LysKB317_EAD-CBD-EAD_) may disrupt enzyme stability and therefore hinder bacteriolytic activity. The DSC findings aligned well with the peptidoglycan binding predictions, and the molecular docking model suggests that proper spatial domain arrangement promotes bacterial cell-wall binding and therefore enhances bacteriolytic performance and enzyme stability.

## 4 Conclusion

Here we demonstrated the feasibility and potential of generating novel endolysin variants through intramolecular domain shuffling of a single parental endolysin, LysKB317. This strategy relies on producing variants with duplicated, flanked, or shuffled domain arrangements. Our findings demonstrated that structural rearrangement alone, without altering domain origin, can significantly impact thermal stability, and bacteriolytic activity of the LysKB317 variants. This endolysin engineering strategy can thus provide a new framework for the rational design of novel Gram-positive bacterial targeting endolysins.

## 5 Materials and methods

### 5.1 Strains and culture conditions

All bacterial and yeast strains are listed in Table 1. Unless otherwise stated, all *E. coli* strains used in this study were grown in tryptic soy broth (TSB; BD Difco) supplemented with the appropriate antibiotic at 37°C with aeration at 200 rpm. All lactic acid bacteria tested were grown in De Man, Rogosa and Sharp (MRS; Difco) brothat 30°C. When required, carbenicillin (Cb; Sigma-Aldrich) at 100 µg/mL, and chloramphenicol (Chl; Sigma-Aldrich) at 34 µg/mL were added. *Saccharomyces cerevisiae* strain NRRL Y-2034 was grown at 30°C with aeration at 100 rpm using yeast extract peptone dextrose media (YPD; Difco) with 5% glucose (v/w; Sigma-Aldrich).

**Table 1:** Strains used in this experiment.

Strains	Relevant genotype or phenotype^a^^,^^b^^,^^c^^,^^d^	Refs. or source^e^
*Escherichia coli*		
BL21(DE3)pLysS/pET21a::LysKB317-EAD	aAmp^R^, bChl^R^, containing LysKB317 EAD domain (C-terminus 6xHis-tag)	This study
BL21(DE3)pLysS/pET21a::LysKB317	aAmp^R^, bChl^R^, containing LysKB317 wild-type (N-terminus 6xHis-tag)	This study
BL21(DE3)pLysS/pET21a::LysKB317-EADx2-CBD	aAmp^R^, bChl^R^, containing two LysKB317 EAD domains fused to N-terminus CBD domain (N-terminus 6xHis-tag)	This study
BL21(DE3)pLysS/pET21a::LysKB317-EADx3-CBD	aAmp^R^, bChl^R^, containing three LysKB317 EAD domains fused to N-terminus CBD domain (N-terminus 6xHis-tag)	This study
BL21(DE3)pLysS/pET21a::LysKB317-EAD-CBD-EAD	aAmp^R^, bChl^R^,containing two LysKB317 EAD domains flanking CBD domain (N-terminus 6xHis-tag)	This study
BL21(DE3)pLysS/pET21a::LysKB317-CBD	aAmp^R^,^b^Chl^R^, containing LysKB317 CBD domain (C-terminus 6xHis-tag)	This study
BL21(DE3)pLysS/pET21a::LysKB317-EAD-CBDx2	aAmp^R^, bChl^R^, containing one LysKB317 EAD with two C-terminus fused CBD domains (N-terminus 6xHis-tag)	This study
BL21(DE3)pLysS/pET21a::LysKB317-CBD-EAD-CBD	aAmp^R^, bChl^R^,containing two LysKB317 CBD domains flanking EAD domain (N-terminus 6xHis-tag)	This Study
BL21(DE3)pLysS	bChl^R^, F- *ompT hsdS*B (rB-mB-) *gal dcm* (DE3) pLysS	Invitrogen
*Limosilactobacillus fermentum*		
0315-1B	cWildtype, biofilm producer	[32]
0315-25	cWildtype	[32]
*Saccharomyces cerevisiae*		
NRRL Y-2034	Ethanol producing strain	dNRRL

a Amp^R^, ampicillin resistant;

b Chl^R^, chloramphenicol resistant;

c Wild-type microbial strain was isolated from a Midwestern dry-grind fuel ethanol facility isolated from a previous screen;

d NRRL also known as the ARS culture collection

**Table 2:** Corn mash fermentation HPLC metabolite analysis at the end of 72 h.

Treatment [0.01 µM]	*L. fermentum* 0315-25 Log(CFU/mL)	Glucose (%)	Ethanol (%)	Acetic acid [M]	Lactic acid [M]
Yeast only control	< 3.0	0.51 ± 0.05	10.44 ± 0.26	0.0104 ± 0.0018	0.0086 ± 0.0009
Yeast + Infection control	8.99 ± 0.01	2.03 ± 0.18	9.21 ± 0.11	0.0190 ± 0.0002	0.0479 ± 0.0003
Yeast + Infection + LysKB317	< 3.0	0.49 ± 0.07	10.30 ± 0.04	0.0106 ± 0.0007	0.0174 ± 0.0004
Yeast + Infection + LysKB317_EADx3-CBD_	< 3.0	0.43 ± 0.01	10.35 ± 0.18	0.0105 ± 0.0009	0.0169 ± 0.0002
Yeast + Infection + LysKB317_CBD-EAD-CBD_	< 3.0	0.42 ± 0.02	10.34 ± 0.09	0.0106 ± 0.0005	0.0193 ± 0.0005

Fermentation flasks with *S. cerevisiae* strain NRRL Y-2034 were grown in corn mash feedstock and challenged with *L. fermentum* 0315-25 at 10^6^ CFU/mL [33] and treated with 0.01 µM purified recombinant LysKB317 variant constructs for 72 h. Enumeration of *L. fermentum* was performed on MRS agar plates with antifungal cycloheximide and corn mash samples were analyzed with HPLC to determine utilization of glucose (%) w/v, production of ethanol (%) w/v, lactic acid [M], and acetic acid [M] generation.

**Table 3:** DSC analysis of LysKB317 variant constructs.

	EAD	EAD-CBD	EADx2-CBD	EADx3-CBD	EAD-CBD-EAD	EAD-CBDx2	CBD-EAD-CBD	CBD
**MW (kDa)**	25.6	33.85	57.04	80.24	57.04	45.31	45.31	12.44
**pI**	5.65	6.05	5.73	5.56	5.73	6.27	6.27	7.95
**T_max_ (°C)**	64.76 ± 0.073	62.78 ± 0.032	60.07 ± 0.026	58.61 ± 0.039	54.44 ± 0.081	62.99 ± 0.015	62.67 ± 0.002	67.12 ± 0.004
**∆H (kJ/mol)**	149.53 ± 21.21	777.69 ± 2.43	420.97 ± 17.37	865.48 ± 12.94	141.11 ± 7.86	1400.53 ± 45.89	1900.07 ± 31.96	179.76 ± 4.08
**∆S (kJ/(mol‧K))**	0.44 ± 0.06	2.32 ± 0.01	1.26 ± 0.05	2.61 ± 0.04	0.43 ± 0.02	4.17 ± 0.14	5.66 ± 0.10	0.53 ± 0.01
**ΔCp (kJ/mol‧K)**	1.87 ± 0.27	10.07 ± 0.03	5.74 ± 0.24	12.16 ± 0.19	2.16 ± 0.12	18.07 ± 0.59	24.66 ± 0.41	2.15 ± 0.05
**Amplitude**	46.86 ± 7.19	224.24 ± 3.06	134.15 ± 3.73	278.36 ± 4.82	37.04 ± 3.06	409.17 ± 20.19	610.93 ± 9.01	27.25 ± 0.53
**FWHM (°C)**	3.02 ± 0.04	3.27 ± 0.03	2.96 ± 0.04	2.95 ± 0.01	3.63 ± 0.18	3.22 ± 0.06	2.90 ± 0.01	6.22 ± 0.03
**Area**	150.60 ± 21.52	779.60 ± 3.57	422.80 ± 16.15	873.70 ± 11.47	142.67 ± 8.15	1403.00 ± 47.00	1887.33 ± 30.86	180.53 ± 4.42

### 5.2 Domain-shuffled constructs

Domain-shuffled endolysins were designed based on LysKB317 (GenBank accession number AIY32273.1; [33]) and were synthesized by GenScript (Fig. 1; GenScript). The engineered variants (LysKB317_EADx2-CBD_, LysKB317_EADx3-CBD_, LysKB317_EAD-CBD-EAD_, LysKB317_EAD-CBDx2_, and LysKB317_CBD-EAD-CBD_) were generated by duplicating and rearranging the enzymatically active domain (EAD) and cell-binding domain (CBD) while retaining the native 18 amino acids interdomain linker (KPGEIKTDTPSAPAPAPK) between adjacent domains. The linker region was identified based on AlphaFold 3 structural prediction [36,61] and the National Center for Biotechnology Information (NCBI) Conserved Domain Database [62,63] to preserve the spacing and flexibility found in the wild-type LysKB317 endolysin.

All domain-shuffled constructs were designed with an N-terminal His_6x_-tag to maintain a consistent cloning, expression, and purification strategy except for the single domain constructs, EAD-only and CBD-only where a C-terminal His-tag was used for consistency without any noticeable effects on protein expression, purification and function. The sequences of all LysKB317 variants (LysKB317_EAD_, LysKB317_EADx2-CBD_, LysKB317_EADx3-CBD_, LysKB317_EAD-CBD-EAD_, LysKB317_CBD_, LysKB317_EAD-CBDx2_, LysKB317_CBD-EAD-CBD_) were deposited into GenBank with accession numbers: PP101025, PP101026, PP101027, PP101028, PP101029, PP101030, and PP101031 in that order.

### 5.3 Expression and purification of recombinant domain shuffled variant endolysins

*E. coli* cells containing variant endolysin constructs (Table 1) were grown overnight in TSB media containing carbenicillin (Cb; 100 µg/mL) at 37°C with aeration. Cells were diluted at 1: 100 ratios with 100 mL of fresh TSB media and supplemented with 100 µg/mL Cb and grown to an optical density (OD_600_ nm) of 0.6 before induction with 0.25 mM of isopropyl β-d-1-thiogalactopyranoside (IPTG; Sigma-Aldrich) at 30°C with 200 rpm shaking for 18 h. Induced cells were harvested by centrifugation at 8,000 × *g* for 15 min at 4°C and lysed with B-PER^TM^ complete bacterial protein extraction reagent following manufacture’s protocol (ThermoFisher). Whole-cell lysate was centrifuged at 15,000 × *g* for 5 min and filter sterilized with 0.22 µm polyethersulfone (PES) membrane syringe filter (Millipore). The filtrate was purified with a prewashed (10 CV volume and equilibrated) TALON® Superflow^TM^ 1 mL column (Cytiva) at 0.1 mL/min using an AKTA pure^TM^ 150 fast protein liquid chromatography (FPLC; Cytiva). The target proteins were eluted in 5-mL fractions with 10 CV of equilibration buffer containing 200 mM imidazole and 10 % glycerol. Sample fractions were analyzed with sodium dodecyl sulfate polyacrylamide gel electrophoresis (SDS-PAGE) using an Any kD TGX precast Tris-glycine acrylamide gels (Bio-Rad) at 110 V for 55 min and visualized using a ChemiDoc XRS+ imaging system (Bio-Rad).

### 5.4 Bacteriolytic assay (BLA)

Bacteriolytic activity was evaluated using a SYTOX Green fluorometric assay adopted from Harhala et al. [64] with modification was utilized to measure the lytic activity of endolysins. The SYTOX Green (Invitrogen) is a membrane impermeable nucleic acid dye that fluoresces upon entering cells with compromised membrane, providing an indirect measure of bacterial lysis. Briefly, the dye was diluted in 1:1000 ratio in citric acid buffer (300 mM NaCl, 30% (v/v) glycerol, 21 mM citric acid, 58 mM NaH_2_PO_4_, pH 5.5). The same buffer was used to prepare the target bacteria (*L. fermentum* 0315-25) and to measure bacteriolytic activity. Cells were grown to OD_600_ = 1 in MRS media, centrifuged (8,000 × *g* for 10 min) and washed twice and resuspended in citric acid buffer to OD_600_ = 1. Positive control cells were washed and further treated in 70 % isopropanol for 1 h at room temperature with mixing at 15 min intervals. In a black clear bottom 96-well plate (Costar), the following samples were assayed in triplicate: buffer control (50 µL of diluted SYTOX Green, 150 µL citric acid buffer), enzyme control (50 µL of diluted SYTOX Green, 50 µL of 100 µg/mL endolysin and 100 µL citric acid buffer), negative control (50 µL of diluted SYTOX Green, 50 µL of OD_600_ = 1 bacteria cells, 100 µL citric acid buffer), and the treatment (50 µL of diluted SYTOX Green, 50 µL of 100 µg/mL endolysin, 50 µL of OD_600_ = 1 bacterial cells, 50 µL citric acid buffer), positive control (50 µL of diluted SYTOX Green, 50 µL of OD_600_ = 1 isopropanol treated bacteria cells, 100 µL citric acid buffer). A microplate reader (SpectraMax ID5, Molecular Devices) was used to read every minute for 120 min at room temperature using a kinetic setting, read from the bottom with 30 flashes per second at 485 nm/535 nm (excitation/emission). All purified endolysin variants were filtered through sterile syringe 0.22 µm PES membrane fileters (Millipore Sigma) and concentration quantified using Qubit Protein assay (ThermoFisher) and diluted to 0.01 µM concentration for bacteriolytic assay (BLA). Fluorescent signals were measured to determine kinetics to compare the bacteriolytic activity of endolysin variants. The initial lytic rate was calculated as BLA with Eq. (1).

(1) $$\begin{array}{c} \mathrm{BLA}=\left({\Delta \mathrm{OD}}_{600}\right)/\left(\left(\Delta T\right)\cdot \left[E\right]\right)=\mathrm{OD}/s\cdot \mu M \end{array}$$

Where ΔOD_600_ is the difference in optical density at 600 nm (OD_600_); ΔT represents the differences between time points in seconds (s) where the time window exhibiting the greatest slope in the early phase of the reaction; and [E] represents the concentration of endolysin in micromolar (µM).

### 5.5 LysKB317 variants analysis using a differential scanning calorimeter (DSC)

A differential scanning calorimeter, NanoDSC with platinum capillary cells (TA Instruments) was used to measure the thermal stability of endolysin variants, [65]. FPLC purified endolysins (described above) and positive control lysozyme from chicken egg white (Sigma-Aldrich) were adjusted to 500 µg/mL in 700 µL assay buffer (300 mM NaCl, 30% (v/v) glycerol, 21 mM citric acid, 58 mM NaH_2_PO_4_, pH 5.5; [33]) that had been degassed at 625 mmHg using Degassing Station (TA Instruments) for 15 min prior to loading the protein samples into capillary cells. A heating ramp rate of 1°C per minute in the temperature range of 25 – 100°C with a total scan for 600 s equilibration was performed. Thermograms were evaluated using NanoAnalyze software version 3.11.0 (TA Instruments). Each protein sample was analyzed in three independent replicates. The change in heat capacity (ΔCp) was estimated using Eq. (2).

(2) $$\begin{array}{c} \Delta \mathrm{Cp}=\left(\Delta H-T_{\max}\cdot \Delta S\right)/T_{\max}=\mathrm{kJ}/\left(\mathrm{mol}\right)\cdot \left(K\right) \end{array}$$

Where ΔH is the change in enthalpy; ΔS is the change in entropy and T_max_ is the thermal unfolding transition temperature. K stands for degree in kelvin.

### 5.6 Corn mash fermentation

The assay was adopted from Patel et al. [66]. Briefly, *S. cerevisiae* strain Y-2034 (Table 1) was grown in YPD media containing 5% glucose (w/v) at 30°C with aeration to mid-log growth phase (OD_600_ = 0.6 – 0.8) and washed with 1x sterile PBS and resuspend to OD_600_ = 1. The target bacterial infection strain *L. fermentum* 0315-25 (Table 1) was grown in MRS with 5% glucose (w/v) at 37°C static to mid-log phase then washed and resuspended to OD_600_ = 4. An OD_600_ of 1 corresponded to approximately 6 × 10^7^ CFU/mL for yeast and 1 × 10^8^ CFU/mL for bacteria. Autoclaved corn mash collected from a commercial dry grind ethanol facility (approximately 33% solids) was treated with glucoamylase (Alcoholase II Liquid) and 0.12 % (w/v) ammonium sulfate ((NH_4_)_2_SO_4_) added for liquefaction at 40°C and 100 rpm agitation for at least 24 h. For each flask, 16 mL of treated corn mash, 0.1 mL yeast inoculum, 0.4 mL of challenged bacterial inoculum, purified endolysin (0.01 µM final concentration) and water were added to a final volume of 20 mL. A rubber stopper was used to plug the flask and a 0-gauge 0.9 mm × 40 mm Precision Glide needle (Becton Dickinson) was inserted to vent gas (CO_2_) generated from fermentation at 30°C with 100 rpm agitation for 72 h. At 0 h, 24 h, 48 h and 72 h, 500 µL samples were taken and diluted in sterile 1 × PBS (pH 7.4) for plating on an MRS agar with 10 µg/mL cycloheximide (Sigma-Aldrich) using the Eddy Jet 2W spiral plater (IUL Instruments) set in the E mode 50 (50 µL sample). Plates were incubated overnight at 37°C and CFU/mL was enumerated using a Flash & Go plate reader (IUL Instruments). Three independent biological replicates were performed for each condition.

### 5.7 Biofilm control assay

The assay protocol was adopted from Rich et al. [67]. Briefly for each assay, 20 µL of overnight *L. fermentum* culture and 150 µL fresh MRS media were pipetted into a Nunc flat bottom polystyrene 96-well microtiter plate (ThermoFisher) and Parafilm was used to seal the plate with a sterile lid to prevent evaporation and incubated at 37°C for 48 h until cells reach static adhesion phase. The lid was removed after 48 h and each well was agitated with a multichannel pipet before a 96-pin replicator lid (Nunc-TSP 96-pin replicator; ThermoFisher) was dipped into the 96-well plate with agitated cells and transferred into a new 96-well plate with fresh 150 µL MRS broth and incubated further at 37°C statically for 48 h. The 96-pin replicator lid was transferred into a new 96-well plate with fresh 150 µL MRS for every 48 h for a total of three times. At the end of the last transfer, purified endolysin LysKB317 treatment at 20 µM, was added into the plate with MRS to a final volume of 150 µL/well. The 96-pin replicator with biofilm was dipped into the treatment plate for 1 h at room temperature. After 1 h of treatment, the 96-pin lid was removed from the treatment plate and washed using sterile ultra-pure water by dipping and lifting the pin lid into a fresh 96-well microtiter plate a few times before drying the 96-pin lid in an Isotemp oven (Fisher Scientific) at 65°C for 30 min. The dried pin lid was stained in 200 µL 0.1% crystal violet solution (BD Difco^TM^) for 30 min at room temperature before washing the pins in fresh 96-well plate with 200 µL sterile ultra-pure water by dipping and lifting. Washed 96-pin lid was dried in an incubator at 37°C for 1 h before destaining the pin lid in a fresh 96-well plate filled with 200 µL/well 95% ethanol for 30 min at room temperature before reading the plate using a SpectraMax ID5 plate reader (Molecular Devices) set to measure endpoint read at OD 540 nm absorbance wavelength [68].

### 5.8 HPLC metabolite analysis

The samples taken at each time point (0, 24, 48 and 72 h) were centrifuged at max speed (≥ 18,000 × *g* for 1 min at room temperature) using a benchtop centrifuge. Two hundred microliters of supernatant were removed for analysis using a high-performance liquid chromatography (HPLC) system (Shimadzu) equipped with a 300 mm Aminex HPX-87H column at 0.5 mL/min, 5 mM H_2_SO_4_ at 65°C (Bio-Rad).

### 5.9 Protein-Ligand Docking Prediction

Structural predictions of domain-shuffled endolysin variants were generated using AlphaFold3 [36,61]. Protein PDB files of the endolysins and a NAG-NAM-NAG-NAM tetrasaccharide polymer as a ligand (PDB number 4PI9 ligands ID 3LT; [54]) were used to simulate docking using High Ambiguity Driven protein-protein DOCKing (HADDOCK version 2.4; [52,53]). The enzyme active site was further predicted using PrankWeb [51].

### 5.10 Statistical analysis

All experiments were performed using three independent biological replicates. Where appropriate, experimental results were analyzed using two-way analysis of variance (ANOVA) with the Tukey post-hoc test to determine significance, where *p < 0.05, **p < 0.01, ***p < 0.001, and ****p < 0.0001 were set to be statistically significant (GraphPad Prism version 9.5.1 and Microsoft Excel 365 version 2503).

## Funding

The research funding was provided by the United States Department of Agriculture, Agricultural Research Service.

## Author disclaimer

Mention of trade names or commercial products in this publication is solely for the purpose of providing specific information and does not imply recommendation or endorsement by the U.S. Department of Agriculture (USDA). The USDA prohibits discrimination in all its programs and activities on the basis of race, color, national origin, age, disability, and where applicable, sex, marital status, family status, parental status, religion, sexual orientation, genetic information, political beliefs, or because all or part of an individual’s income is derived from any public assistance program. The USDA is an equal opportunity provider and employer.

## CRediT authorship contribution statement

**Shao-Yeh Lu:** Writing – review & editing, Writing – original draft, Methodology, Formal analysis, Data curation, Conceptualization. **Zhengliang L. Wu:** Writing – review & editing, Investigation. **Moses Y. Martinez:** Writing – review & editing, Methodology, Formal analysis. **Maulik H. Patel:** Writing – review & editing, Methodology, Conceptualization. **Christopher D. Skory:** Writing – review & editing, Methodology, Investigation, Conceptualization.

## Declaration of competing interest

The authors declare no conflicts of interest.

## Data Availability

Data will be made available on request.

## Appendix Supplementary materials

Image, application 1
