# Supplementary material for: Domain shuffling of endolysin LysKB317 to control Limosilactobacillus fermentum in corn mash fermentation
Source: Biotechnol Rep (Amst). 2026 Jul 19;51:e00973. doi: 10.1016/j.btre.2026.e00973 (PMC13445470; doi:10.1016/j.btre.2026.e00973)
Supplement: Supplementary file 1 [file mmc1.docx]

**SUPPLEMENTAL MATERIALS**

**Domain Shuffling of Endolysin LysKB317 to Control *Limosilactobacillus fermentum* in Corn Mash Fermentation**

Shao-Yeh Lu^1*^, Zhengliang L. Wu^1^, Moses Y. Martinez^1^, Maulik H. Patel^2^ and Christopher D. Skory^1^

^1^USDA, Agricultural Research Service, National Center for Agricultural Utilization Research, Renewable Product Technology Research Unit, Peoria, IL 61604, USA

^2^Oak Ridge Institute for Science and Education (ORISE), Oak Ridge, TN 37830, USA

*Corresponding author:

Shao-Yeh Lu, Ph.D., United States Department of Agriculture, Agricultural Research Service, National Center for Agricultural Utilization Research, Renewable Product Technology Research Unit, 1815 N. University St., Peoria, IL 61604-3902, USA; +01 (309) 681-6067; shao.lu@usda.gov

**Supplemental Figure**

**Figure S1.** Predicted protein tertiary structures of the domain shuffled endolysin LysKB317 constructs utilizing AlphaFold 3 [36, 61].

**Figure S2. Bacteriolytic activity (BLA) of individual domains and domains swapped variants of LysKB317.** Treatments: Cell-wall binding domain (CBD), enzymatically active domain (EAD), LysKB317_CBD-EAD-CBD_ (CBD-EAD-CBD), LysKB317_EAD-CBD-EAD_ (EAD-CBD-EAD), LysKB317_EADx2-CBD_ (EADx2-CBD), LysKB317_EAD-CBDx2_ (EAD-CBDx2), LysKB317_EADx3-CBD_ (EADx3-CBD), and LysKB317 wild-type (WT). Data are reported in relative fluorescent units per second micromolar (RFU/(s‧µM)). Three independent replicates (*n* = 3); error bars indicate standard deviation (SD).

**Figure S3. LysKB317 ligand binding site and docking site prediction.** Webserver PrankWeb [51] and HADDOCK2.4 [53] were utilized to predict binding and docking of peptidoglycan. (A). Highlighted 3 predicted binding pockets visualization of LysKB317 (B). Cell-wall binding domain (CBD) posterior view of predicted binding pocket. (C). Highlighted region of central predicted pocket view of LysKB317. (D). Anterior view of predicted binding pocket of enzymatically active domain (EAD). (E). Surface protein visualization of 3 predicted ligand binding sites of LysKB317. (F). Predicted docking site of using a NAG-NAM-NAG-NAM tetrasaccharide polymer as a ligand (PDB number 4PI9 ligands ID 3LT; [54]) to simulate peptidoglycan fragment (red) in cartoon and molecular surface representation.

**Figure S4.** **Bacterial lytic activity at room temperature (22℃) and heat treated (63℃) endolysin variants measured via SYTOX assay.**
